# Four genes essential for recombination define GInts, a new type of mobile genomic island widespread in bacteria

**DOI:** 10.1038/srep46254

**Published:** 2017-04-10

**Authors:** Leire Bardaji, Myriam Echeverría, Pablo Rodríguez-Palenzuela, Pedro M. Martínez-García, Jesús Murillo

**Affiliations:** 1 Departamento de Producción Agraria, Escuela Técnica Superior de Ingenieros Agrónomos, Universidad Pública de Navarra, 31006 Pamplona, Spain; 2 Centro de Biotecnología y Genómica de Plantas, E.T.S. Ingenieros Agrónomos, Universidad Politécnica de Madrid, Campus de Montegancedo, E-28223 Pozuelo de Alarcón, Madrid, Spain; 3 Instituto de Hortofruticultura Subtropical y Mediterránea “La Mayora”, Universidad de Málaga-Consejo Superior de Investigaciones Científicas (IHSM-UMA-CSIC), Área de Genética, Facultad de Ciencias, Campus Teatinos s/n, 29010 Málaga, Spain

## Abstract

Integrases are a family of tyrosine recombinases that are highly abundant in bacterial genomes, actively disseminating adaptive characters such as pathogenicity determinants and antibiotics resistance. Using comparative genomics and functional assays, we identified a novel type of mobile genetic element, the GInt, in many diverse bacterial groups but not in archaea. Integrated as genomic islands, GInts show a tripartite structure consisting of the *ginABCD* operon, a cargo DNA region from 2.5 to at least 70 kb, and a short AT-rich 3′ end. The *gin* operon is characteristic of GInts and codes for three putative integrases and a small putative helix-loop-helix protein, all of which are essential for integration and excision of the element. Genes in the cargo DNA are acquired mostly from phylogenetically related bacteria and often code for traits that might increase fitness, such as resistance to antimicrobials or virulence. GInts also tend to capture clusters of genes involved in complex processes, such as the biosynthesis of phaseolotoxin by *Pseudomonas syringae*. GInts integrate site-specifically, generating two flanking direct imperfect repeats, and excise forming circular molecules. The excision process generates sequence variants at the element attachment site, which can increase frequency of integration and drive target specificity.

Tyrosine-based site-specific recombinases (TRases) are exceedingly abundant in prokaryotic and eukaryotic genomes^1^,^2^, carrying out functions as diverse as ensuring proper chromosomal and plasmid segregation, regulating gene expression or determining the life cycle of prophages towards dormancy or cell death^3^. They are also responsible for the movement of DNA through various types of mobile genetic elements (MGEs), such as integrons, phages, certain transposons, integrative conjugative elements, and genomic islands^1^,^4^. These elements have a significant impact in bacterial evolution by spurring lateral gene transfer, which favours the speciation of bacteria and their adaptation to new ecological niches, either by enhancing their fitness and/or enabling interactions with other organisms^4^,^5^,^6^. For instance, integrons are the major agents for the dissemination of antibiotic multiresistance in Gram-negative bacteria^7^, whereas integron-like elements are of significance to the transfer of fitness and virulence genes among environmental bacteria^8^. Likewise, genomic islands are key players in microbial evolution and adaptations that are of medical, agricultural and environmental interest^9^,^10^; indeed, many plant and animal pathogenic bacteria carry diverse genomic islands that allow them to more efficiently colonize their hosts and evade control measures, threatening the management of diseases in human settings^11^.

The bean plant pathogen *Pseudomonas syringae (Ps*) pv. phaseolicola contains a cluster of 23 genes, the Pht cluster, responsible for the biosynthesis of the wide-spectrum antimetabolite toxin phaseolotoxin^12^,^13^,^14^. Besides its relevance as a research model and potential biotechnological applications^15^, the Pht cluster is of practical significance because it is generally used for the specific detection of this important plant pathogen^16^. The cluster is embedded into the 38 kb putative pathogenicity island Pht-PAI, whose 5′ end is defined by three genes coding for products with similarities to TRases^13^,^17^. The Pht-PAI appears to be mobile, because a nearly identical genomic island has been found syntenically inserted into the genome of certain strains of the kiwiplant pathogen *Ps* pv. actinidiae^13^,^17^. Additionally, homologues of the three putative TRase genes were found in *Ps* pv. tomato DC3000 and *Ps* pv. phaseolicola CYL314, integrated into the same genomic location as the Pht-PAI but associated to dissimilar DNA^13^,^17^. These correlational data, together with the phylogeny of diverse genes from the Pht cluster, led to the hypothesis that this putative island was possibly first acquired from a Gram-positive bacterium by lateral gene transfer, and later exchanged among *P. syringae* pathovars^13^,^17^,^18^. Nevertheless, there is a paucity of functional and structural data demonstrating the existence of this putative mobile genomic island, and for assessing its potential impact for the dissemination of virulence genes among *P. syringae* and other bacteria.

Here, we used comparative genomics and functional assays to study the functionality of the Pht-PAI and its potential mobility. This showed that the Pht-PAI belongs to a new type of MGE, designated GInt, that is widely distributed in diverse bacterial phyla. GInts are characterized by the *ginABCD* operon, coding for three TRases and a fourth product that are essential for recombination, and can carry up to at least 70 kb of cargo DNA containing genes potentially increasing fitness and virulence.

## Results

### GInts are a new type of genomic island

The Pht-cluster is included in a genomic island (Pht-PAI) that has been exchanged horizontally among *Ps* strains, and it is accompanied by three putative TRases (here named genes *ginABC*) that are associated to dissimilar DNA in different strains^13^,^14^,^19^. We therefore postulated that these putative integrases might then be part of a functional mobile element carrying different cargo DNA.

**Figure 1:**
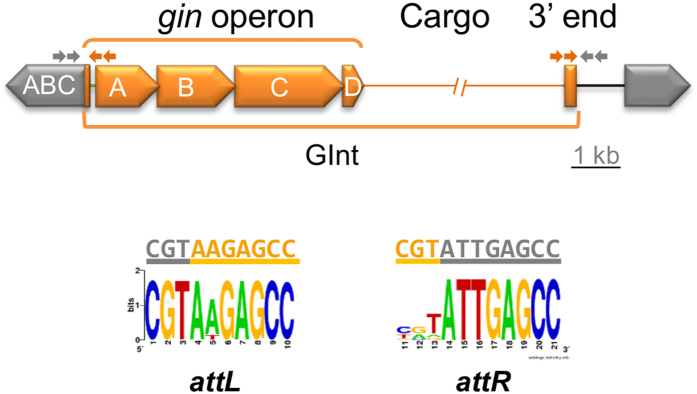
General structure of GInts. The GInt element (orange, with bacterial sequences in grey) has a tripartite structure: The highly conserved *ginABCD* operon, the cargo DNA (broken line), and a 0.2–0.4 kb poorly conserved 3′ end. GInts integrate site-specifically; the GInt carrying the Pht-PAI from *P. syringae* pv. phaseolicola 1448A, and related elements, integrate within a putative ABC transporter gene (PSPPH_4293 in strain 1448A; indicated as ABC). Small grey and orange arrows represent primers for testing excision and circularization. The site-specific integration of GInts generates two direct imperfect repeats (*attL* and *attR*) of 10 and 11 nucleotides, which are chimeras of the *attI* sequence from the GInt (orange letters) and the *attB* sequence from the bacterial chromosome (grey lettering). The sequence logo was generated from alignments of the *att* repeats from GInts related to the Pht-PAI (Table S1).

Using blastn, we found many examples among pseudomonads of genomic islands showing the same organization than the Pht-PAI, namely, a tripartite structure consisting of 1) the highly conserved *ginABCD* operon in the 5′ end; 2) a variable amount of cargo DNA, starting immediately before or after the stop codon of *ginD*; and 3) a short and poorly conserved 3′ end containing some well-conserved AT-rich sequence stretches (Fig. 1 and Table S1). All these islands inserted into the same chromosomal location, the 5′ end of an ABC transporter gene (PSPPH_4293 in strain 1448A) and are flanked by 10–11 nt direct imperfect repeats, the *attL* and *attR* (Fig. 1 and Table S2). Since they show characteristics of novel MGEs (see below), we have collectively designated these elements GInts (Genomic Island with three Integrases),

### Structural and transcriptional analysis of genes *ginABCD*

The alleles of genes *ginA* and *ginB* from 16 analysed genomes (Table S1) showed significant matches with the catalytic core of phage integrases (families IPR011010, IPR013762 and IPR002104), whereas the alleles of *ginC* contain domains matching families IPR011010 and IPR013762, or only IPR013762. The structure of the deduced products from *ginABC* could be modelled using Phyre2, with CRE, XerD or XerC as the closest matching templates. Additionally, Psi-blast multiple alignments of 1000 proteins identified highly conserved residues and the RHRHY motif necessary for integrase function^20^, although the first arginine is missing in GinC (Fig. S1). No domains were found in GinD using InterProScan, although the Phyre2 structure of the C-terminal end of GinD displays an HLH domain.

**Figure 2:**
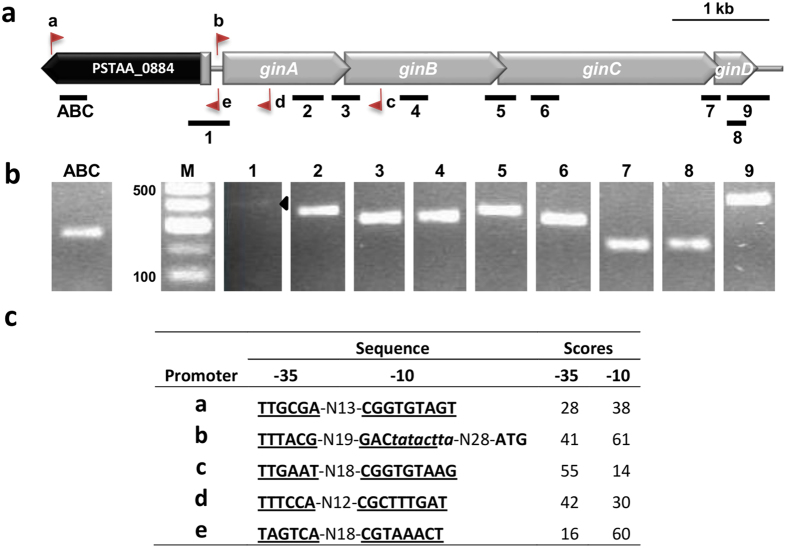
RT-PCR analysis of the *gin operon* from *P. stutzeri* DSM 4166. (**a**) Location of the amplicons observed in RT-PCR experiments. The location and orientation of promoters predicted using the BPROM software is indicated with flags named from a to e. (**b**) Gel electrophoresis of amplicons from RT-PCR experiments generated using strand-specific primers for each amplicon for cDNA synthesis, as shown in panel a; the black triangle in lane 1 identifies a repetitively faint amplification band. The RT-PCR negative controls produced no amplicons, and are not shown for clarity. M, molecular weight marker in nt. (**c**) Sequence and score values of −35 and −10 boxes (underlined) of promoters a to e in panel **a**, as predicted by the BPROM software. A putative Fis binding box is indicated in italics.

RT-PCR analyses showed genes *ginABCD* from strain *Pst* DSM 4166 (Fig. 2A) to be cotranscribed in a polycistronic mRNA, with transcription likely continuing into the cargo DNA (Fig. 2B). Using strand-specific primers for cDNA synthesis, RT-PCR revealed transcription from outside the element towards *ginA*, and from within *ginA* towards the ABC transporter (Fig. 2B). The software BPROM predicts appropriate consensus promoters for all these transcripts (Fig. 2C). These results suggest that genes *ginABCD* constitute an operon that is transcribed from a promoter located within the GInt, upstream of *ginA*, and from an external bacterial promoter. They also indicate that insertion of the GInt directs the transcription of genes situated 5′ of the element.

### GInts excise generating circular intermediates

The mobilization of DNA by integrases involves its excision and the formation of circular intermediates^21^,^22^; we therefore tested by nested-PCR the ability of different GInts to form circular structures and to restore an empty *attB* site in the chromosome (Fig. 1 and Table S2).

**Figure 3:**
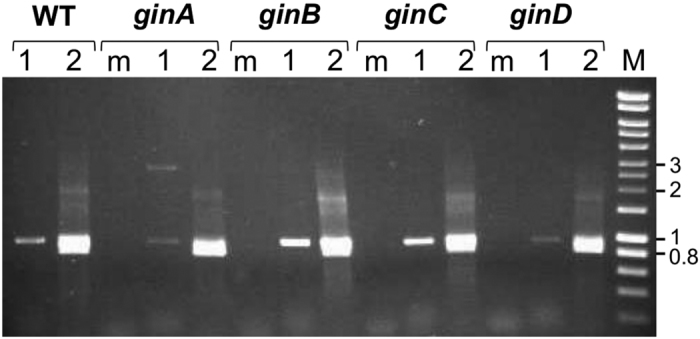
All four *ginABCD* genes are required for insertion and excision of the GInt. Generation of a specific amplicon for the circular intermediate of the GInt from *P. stutzeri* DSM4166, from mutants in each of the *ginABCD* genes (strains UPN821, -823, -825 and -828), and from their corresponding derivatives complemented *in trans* with an intact copy of the mutated *gin* gene. Gel electrophoresis of amplicons generated in the first (1) or second (2) round of nested PCR analyses for the wild type and complemented mutant strains, or in the second round for mutant strains (m). M: HyperLadder I molecular marker (Bioline, Singapore), in kb.

**Figure 4:**
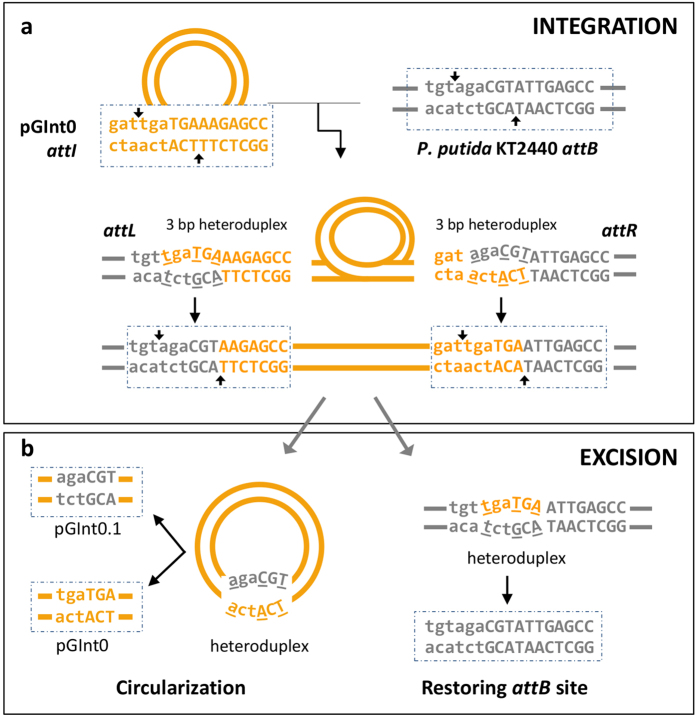
Proposed mechanisms for the general integration and excision of GInts. As a model that fits the observed sequence changes in circular molecules (see Table S2), the cartoon shows the integration and excision of the artificial GInt pGInt0 (orange) into the genome of *P. putida* KT2440 (grey), with boxed sequences observed by sequencing of reaction products. (**a**) Gin proteins likely catalyse 6 nt staggered cuts (vertical arrows) at both *attI* and *attB* sites, resulting in the generation of hybrid *attL* and *attR* sites upon integration of pGInt0; these are resolved in the cell likely by replication or mismatch repair. Heteroduplexes are shown as curved sequences with differing nucleotides underlined. (**b**) Excision of the element likely involves 6 nt staggered cuts within *attL* and *attR* sites (shown in panel a), with the concomitant circularization of pGInt0 and generation of hybrid restored *attI* and *attB* sites. Repair of heteroduplexes might recreate the original *attI* and *attB* sequences or introduce nucleotide changes, depending of which sequence (from the GInt or from the bacterium) is used as template for repair. The generation of alternative *attB* sites upon excision was not observed in the limited sample sequenced from strain KT2440, but it occurred after excision of GInts in other strains (Table S2).

The second round of nested PCR amplifications generated the expected amplicons for the circular intermediate for all tested bacteria (Table S2), although the low PCR yield suggests that it occurs in only a few cells. Additionally, we detected the amplicon for *Ps* pv. phaseolicola 1448A in the rich medium KB at all time points examined (see Methods), but not in minimal medium or *in planta.* Remarkably, for *Pst* DSM 4166 the specific amplicon was already detectable in the first round of PCR (Fig. 3), suggesting that the circularization of the GInt is more frequent in this strain. Similarly, we were able to detect the chromosomal scar corresponding to an empty *attB* site only in cultures of *Ps* pv. pisi 1704B, *P. cannabina* pv. alisalensis ES4326 and *Ps* pv. tomato DC3000 (Table S2), but not for the remaining strains. These unexpected results suggest that the excision of certain GInts might be replicative, leaving a copy into the *attB*; might be lethal for the bacterium; or might induce genetic changes in the insertion site. DNA sequence of cloned PCR products for GInt junctions and scars from six different bacteria was as expected by recombination at the defined *attL* and *attR* sites, thus confirming the excision of the GInts and the formation of a circular intermediate (Table S2). However, and because the *attR* and *attL* sequences are not identical, we observed the reconstruction of different *attI* sites, some of which appeared to have incorporated a few nucleotides from the bacterial chromosome (Fig. 4 and Table S2). This is compatible with excision of the GInt involving 6 nt staggered cuts and further resolution of 3 nt heteroduplexes by replication or mismatch repair^23^. Likewise, we observed restored *attB* sites from *Ps* pv. pisi 1704B and *P. cannabina* pv. alisalensis ES4326 differing from the sequence in related bacteria and likely resulting from a resolution of the heteroduplex towards the *attI* sequence (Fig. 4 and Table S2). We also found deletions of 2 or 6 nt in other cloned *attB* sites from strain 1704B, although we do not have a satisfactory explanation for their occurrence.

### GInts are exchanged interspecifically

*Ps* pv. phaseolicola and *Ps* pv. actinidiae contain nearly identical Pht-PAI islands, although they are assigned to different species^13^,^17^,^24^, suggesting that GInts are mobilized among bacterial populations. However, despite numerous attempts, we were unable to demonstrate the transfer of GInts from *Ps* pv. phaseolicola 1448A and *Pst* DSM 4166 to strains of *Ps* pv. syringae (see Methods), indicating that either our experimental conditions were not conducive for transfer or that its frequency is lower than our detection threshold.

To examine horizontal mobility, we compared the phylogenies of GInts and host strains. As expected, the phylogeny of *ginA* (Fig. S2), and remaining *gin* genes (data not shown), was not congruent with the MLST-based phylogeny for bacterial strains^24^,^25^, indicating that GInts are frequently subjected to horizontal transfer.

### Functional analysis of GInts

To perform functional studies of these mobile elements we synthesized pGInt0, a minimal self-contained GInt derived from *Pst* DSM4166 and cloned in a suicide vector, which serves as cargo DNA and confers Km^r^.

We were able to transfer pGInt0, by electroporation, to strains *P. fluorescens* SBW25, *P. putida* KT2440 and *Ps* pv. syringae UMAF0158, routinely obtaining up to 1.1 ± 0.5 × 10^3^ Km^r^ transformants μg^−1^ DNA. PCR analysis of at least 10 independent Km^r^ clones per strain indicated that, as expected, pGInt0 integrated site-specifically into the ABC transporter homolog (locus_tag PFLU5327, PP_0674, and PSYRMG_12240, respectively), occupying the previously empty *attB* site and producing the chimeric *attL* and *attR* sequences (Fig. 4 and Table S2). These results demonstrate that pGInt0 serves as a closed, minimal circular intermediate substrate, with all the genetic information needed for integration. PCR amplification of several independent clones of these three strains generated the specific amplicon for the circular molecule in all cases, but the chromosomal scar only in clones from *P. putida* KT2440 and *Ps* pv. syringae UMAF0158. Additionally, cell lysates from these transformants routinely yielded thousands of Km^r^ colonies upon electroporation into *E. coli*, and the resulting clones contained autonomous plasmids with EcoRI and AccI profiles identical to those of pGInt0. These results suggest that pGInt0 is able to integrate and further excise from the chromosome as a circular intermediate.

From our previous results (Table S2), we expected that the pGInt0 excision process would lead to changes in reconstructed *attI* and *attB* sites. Therefore, from six independent clones of the three *Pseudomonas* species, we sequenced four cloned scars from *P. putida* KT2440 and *Ps* pv. syringae UMAF0158 and 22 cloned *attI* sequences. In all cases, the sequence of the scar was identical to the wild type sequence (Fig. 4). Conversely, the sequence of the *attI* was identical to that of pGInt0 in only 50% of the recovered pGInt0 clones, whereas the remaining clones contained the putative 6 nt overhang identical to the *attB* (Fig. 4 and Table S2). One of these plasmids was retained and designated pGInt0.1, containing three nt changes and making the *attI* more similar to the bacterial *attB*. We observed a tenfold increase in the number of Km^r^ transformants μg^−1^ of *Ps* pv. syringae UMAF0158 when using pGInt0.1 (13.7 ± 2.9 × 10^3^) as compared with pGInt0 (1.1 ± 0.5 × 10^3^), indicating that the degree of identity between *attB* and *attI* determines, at least in part, the frequency of integration.

### A complete functional *gin* operon is required for the excision/integration process of GInts

Their high conservation across bacteria (see below), suggests that all *ginABCD* genes participate in the mobilization of GInts. We thus evaluated the ability of mutants in each *gin* gene for excision and integration in, respectively, *Pst* DSM 4166 and pGInt0. Unlike with the wild type strain, we were not able to detect circular intermediates in nested PCR analyses with individual mutants of *Pst* DSM 4166 in each of the *ginABCD* genes (Fig. 3). In complementation assays, we detected the circular intermediate in the first round of nested PCR for all the complemented mutants, indicating that circularization capacity was restored (Fig. 3). Likewise, transformation of *Ps* pv. syringae UMAF0158 with mutant plasmids pGInt0A, -B, -C and -D did not yield any Km^r^ clone, although the complemented mutants yielded 10 to 80 Km^r^ transformants μg DNA^−1^. Together, our results indicate that all four genes from the *ginABCD* operon are essential for excision and integration of GInts.

### Bacterial species and strains contain diverse families of GInts

Blastp comparisons soon indicated that bacterial species could contain two or more *ginABCD* operons showing low sequence identity. For instance, the GInt containing the Pht-PAI from *Ps* pv. phaseolicola 1448A (Table S1) and those found in *Ps* pv. syringae UMAF0158 and *Ps* pv. myricae AZ84488 (Table S3) likely define three different evolutive families, because their GinABCD deduced products show only 24–40% identity in pair comparisons. Remarkably, and despite their low relatedness, the GInts in the last two strains are inserted into the same genomic location, at the end of a single stranded DNA-binding protein (*ssb*) gene.

Importantly, the presence of a GInt in a genome does not preclude the acquisition of other GInts that might insert into different genomic locations, and we found frequent instances of strains containing at least two GInts. For example, *Ps* pv. phaseolicola 1448A contains the Pht-PAI and it also contains two other *ginABCD* operons (PSPPH_2793-2796 and PSPPH_3741-3744), both with truncations, which are associated to DNA that is not syntenic in related bacteria. However, they are also associated to other mobile elements that hamper the identification of the corresponding GInt element. Likewise, *P. aeruginosa* PA_D16 also contains two *ginABCD* operons (A6695_20385-20400 and A6695_21815-21830), which are part of two nearly identical GInts of 40.3 and 35.8 kb that are present in a variety of *P. aeruginos*a strains. They also show high insertion specificity, and are inserted at the end of a duplicated ribonucleoside-diphosphate reductase subunit alpha (A6695_20380 and A6695_21835). In all, our results indicate that bacteria are colonized by a variety of GInts, which can insert at different genomic locations and that do not appear to limit the acquisition of other related or unrelated GInts.

### GInts are widely distributed in bacteria

BlastP comparisons of the individual *ginABCD* products retrieves hundreds of hits from diverse groups of bacteria, the majority of which show less than *ca.* 35% amino acid identity with the query; although this suggests a potentially wide distribution of these genes, it does not necessarily imply the existence of GInts in other bacteria. To confirm this, we investigated the presence of the *gin* operon in complete sequenced genomes, because this operon is a distinctive feature of GInts and because other structures of the element are only poorly conserved.

Using the *gin* operon from *Pst* DSM 4166 as a query sequence (see Methods), we retrieved many putative homologues distributed among Alpha-, Beta- and Gammaproteobacteria as well as from the closed genome of the Verrucomicrobia *Opitutus terrae* PB90-1 (see examples in Table S3). Using the *ginABCD* products from *Opitutus terrae* as a blastp query, we found further examples of *gin* operons in the Phyla Bacteroidetes and Firmicutes (examples in Table S3). Although we have not tried to be exhaustive in our search, these results clearly show that the *ginABCD* operon is widely distributed among very different bacterial phyla. Indeed, it is feasible that this operon (and, hence, GInts) might appear in other bacterial groups that we are not detecting because of the inherent limitations of our search, derived in part from the low sequence conservation of the *gin* operons.

From all these *gin* operons, we chose eleven from different species to analyse the existence of identifiable GInts (Table S3). As deduced from genome comparisons among phylogenetically related strains, the *gin* operon was part of a GInt, associated to a cargo DNA of variable size (2.5–70 kb), and was flanked by direct imperfect repeats of 10–17 nucleotides. Six of these GInts were integrated within the same *ssb* gene homolog (Table S3), with the *attL* overlapping the end of the gene without altering the corresponding product. This is remarkable because the amino acid identity levels among their corresponding GinABCD sequences, in Blast comparisons, are lower than 35%, indicating that distantly related GInts might show identical insertion specificity.

### GInts contain very diverse cargo DNA

To investigate the origin of the cargo DNA, we analysed 20 complete GInts, 13 of which are from pseudomonads (Tables S1 and S4). The amount and type of DNA found is highly variable (Table S4), suggesting that GInts are very dynamic elements. In general, the closest homologues for the majority of genes in the cargo DNA was found in strains from the same or related species (Fig. S3, Table S4), indicating that GInts capture genes preferentially from phylogenetically related bacteria. The cargo DNA consists mainly of complete CDSs that in many cases were found in groups of three or more, in regions that were syntenic in other bacteria, conferring specific functions (Table S4). Thus, the acquisition of a GInt could potentially associate to an adaptive advantage for its bacterial host. Some examples of relevant types of cargo DNA are virulence genes such as the T3SS effector genes *hopAZ1* (locus_tag PMA4326_RS23760) and *hopBD1* (locus_tag PMA4326_RS23770), from the crucifer pathogen *P. cannabina* pv. alisalensis ES4326, which likely participate in virulence and modulate host specificity^26^. Another type are gene clusters conferring resistance to antibacterials, including aminoglycosides, quaternary ammonium compounds, chloramphenicol, trimethoprim, tetracycline, sulphonamides, copper, and mercury, such as those found in the important human pathogen *P. aeruginosa* (Table S4). The Pht-PAI is an extreme example of GInts acquiring complex cargo DNA, containing a cluster of 22 genes involved in the biosynthesis of the modified tripeptide phaseolotoxin^14^ (Table S4). Addiction modules, such as DNA restriction and modification systems (Table S4), were also often found in these elements likely favouring their fixation in the host genome. These data indicate that the cargo DNA of GInts may carry out complex functions that would probably allow for the quantum-leap evolution of the acquiring bacteria.

## Discussion

Here we described a novel type of mobile genetic element, the GInt, defined by a four-gene operon (*ginABCD*), coding for three TRases and a hypothetical protein, all of which are essential for mobilization. GInts are inserted as genomic islands of up to at least 76 kb in the genomes of many bacterial groups, showing a high specificity of insertion; they excise as circular intermediates, preferentially distributing within the harbouring bacterial clade, and often contain genes coding for adaptive characters, such as antibiotic resistance or virulence, and clusters of genes involved in complex phenotypes.

GInts display a tripartite structure, with the *ginABCD* operon delimiting the 5′ end as their defining characteristic feature (Fig. 1). The deduced products of *ginABC* contain typical structural and catalytic domains of TRases (Fig. S1), although GinC lacks some of them and it is often annotated in genomes as a hypothetical protein. Gene *ginD* does not show any conserved domain in an InterPro search, but modelisation with Phyre2 predicts a possible helix-loop-helix C-terminal domain. However, GinD shows low but significant identity with TnpC (not shown), which stimulates integration and determines orientation of the site-specific transposon Tn*554* and similar elements^27^,^28^; therefore, it is plausible that GinD might carry a similar function during the integration of GInts. Nevertheless, given the low frequency of insertion of GInts, it would be challenging to show whether or not GinD displays a similar behaviour to that of TnpC. Most other MGEs usually code for only one or two integrases, although for recombination they might require the participation of host factors, such as IHF and Fis^3^, which can carry a variety of functions. Consequently, GInts are the paradigm of a new type of MGE requiring for their mobility three integrases and an additional product that are encoded for by the element. This supports the idea that the involvement of several adjacent integrases for function might be more common than previously thought, because elements containing integrases arranged in tandem or trios are predominant in certain proteobacteria^1^,^8^,^29^,^30^. As it is common for other MGEs^5^, it is feasible that one or more of the integrases from the *ginABCD* operon also participate in the acquisition of foreign DNA by GInts, although there is currently no empirical evidence for this function.

The 3′ terminal end of GInts is only poorly conserved, and we did not identify any common feature that could be involved in self-recognition for integration and excision. However, all elements are bordered by short (at least 10–11 nt) imperfect repeats (*attL* and *attR*; Fig. 1) that are hybrid sequences from the GInt *attI* and the bacterial *attB* (Fig. 4). The excision of GInts appears to follow the general process of other elements containing TRases^23^, involving 6–7 nt staggered cuts at the end of the terminal repeats and circularization of the element. Because the *attL* and *attR* are not identical, this generates a heteroduplex that is further repaired, leading to sequence changes in a fraction of the excised molecules (Fig. 4). An immediate consequence of this is that the *attI* shows a higher sequence identity to the bacterial *attB,* and we showed that these changes (Table S2) led to an increase of the integration frequency of pGInt0 by an order of magnitude. Therefore, we predict that the integration and excision processes by themselves will ultimately direct the evolution of GInts to a higher host specialization, as we have observed for extant elements. This could also explain the tendency of GInts to capture DNA from bacteria phylogenetically related to its host, as they would preferentially be transferred amongst them.

We demonstrated that GInts excise generating a circular intermediate molecule (Figs 3 and 4; Table S2), although with a low frequency. Nevertheless, the excision of the GInt from *Pst* DSM4166 cultures could be detected in the first round of a nested PCR (Fig. 3), suggesting that certain bacterial strains could potentially transfer their GInts with a higher frequency than others. Remarkably, we did not detect episome formation in *Ps* pv. phaseolicola 1448A grown in minimal medium or from populations recovered from bean pods. This was unexpected, because the transfer of diverse mobile genetic elements occurs at higher frequencies during the interaction of several pathogens with their eukaryotic hosts^31^,^32^,^33^. However, it is possible that circularization does occur *in planta*, but at a frequency below the detection limits of our experimental setup. Our phylogenetic analyses (Fig. S2) and previous works^12^,^13^ clearly established that GInts are being horizontally transferred among bacterial populations. However, we were unable to demonstrate the horizontal transfer of the Pht-PAI from *P. syringae* pv. phaseolicola 1448A and the GInt from *Pst* DSM 4166 to two strains of *P. syringae* pv. syringae, either *in vitro* or during the interaction with a plant host. The horizontal transfer of genomic island PPHGI-1 from *P. syringae* pv. phaseolicola occurs by transformation *in planta*, but not *in vitro*, and with a very low frequency^31^. If transformation frequencies of GInts are similar, it is possible that they occur only rarely, mostly taking into account their low excision frequencies. Another possibility is that GInts are mobilized by transduction, as it occurs with many other genomic islands^34^,^35^.

In general, GInts appear to have a very high insertion specificity, and we identified only a few genomic locations where they could be found. Interestingly, several unrelated GInt elements insert either at the beginning (e.g., *Chrysobacterium artocarpi* UTM-3) or, preferentially, at the end of an *ssb* gene (Table S3). This is remarkable given the very low identity among these GInts and among the different *ssb* homologs, and suggests that the signals for recognition of the insertion sites, both in the GInt and in the genome, might be highly conserved. Additionally, insertion of a GInt probably does not cause the complete inactivation of the target genes. In certain cases, GInts localize to intergenic regions or at the 3′ end of genes, leading to a change of variable length in the C-terminal end of the corresponding product. In other cases, they insert at the 5′ end of the target gene, predictably preventing its expression. However, we have shown that the GInt from *Pst* DSM 4166 contains a promoter within the *gin* operon and directed outwards, leading to the expression of the ABC transporter gene located 5′ of the GInt (Fig. 2), and it is likely that this is a general feature of GInts. Indeed, certain other MGEs contain promoters that activate surrounding genes^36^, which can minimize mutational damage and also facilitate adaptability by promoting gene expression^37^.

GInts carry a variable amount of cargo DNA, with three significant properties: it originates mostly from phylogenetically related species, it can incorporate a large amount of contiguous DNA, and often includes post segregational killing systems that may favour their maintenance, as it also occurs with plasmids and other MGEs^38^. The analysis of GInts from *Pseudomonas* related to that containing the Pht-PAI, showed that more than 50% of the cargo DNA of each element had the closest homolog in this bacterial group (Fig. S3). This is not surprising if we take into account that GInts also tend to distribute preferentially within their harbouring bacterial group, a behaviour that is common to other MGEs, such as plasmids^39^ and insertion sequences^40^. We do not have a satisfactory explanation for the tendency of GInts to capture large stretches of DNA, which often code for complex functions such as the biosynthesis of phaseolotoxin^14^, allowing for the quantum-leap evolution of the acquiring bacteria and favouring the acquisition of GInts. GInts might also acquire DNA that is particularly suitable for the hosting bacterium; for instance, we have found GInts carrying several genes for resistance to antibiotics in *P. aeruginosa*, one of the most relevant bacterial human pathogens.

Blastp searches revealed the presence of GInts in very different bacterial phyla, including Bacteroidetes, Firmicutes, Verrucomicrobia and Proteobacteria (Table S3). Our search did not attempt to be exhaustive, and was likely limited by the very low conservation of GInt sequences across bacterial groups; it is therefore likely that GInts have a wider distribution in Bacteria, although we did not find evidences for their presence in Archaea. This wide distribution, together with their ability to carry large DNA fragments coding for complex functions and adaptive characters, make GInts relevant agents in the horizontal distribution of genes and the evolution of bacteria.

## Methods

### Strains and growing conditions

Bacterial strains and plasmids are described in Table S5. *E. coli* was cultured at 37 °C in Luria Bertani medium and *Pseudomonas* strains at 25–28 °C in either King’s medium B (KB) or mannitol-glutamate (MG) agar, which does not support the growth of *E. coli*. When necessary, media were amended with supplements at the following final concentrations (in μg mL^−1^ or % w/v): ampicillin, 100; kanamycin, 50; gentamicin, 10; spectinomycin, 200; streptomycin, 50; sucrose, 5%, and arabinose, 0.5%. Selection for copper resistance was carried out in medium MG plus CuSO_4_·5H_2_O at a final concentration of 200 μg mL^−1^ (0.8 mM).

### Standard molecular techniques

Genomic DNA was purified using a DNA isolation kit (Jet Flex, Genomed, Germany). For sequencing, amplicons were purified using the PCR Extract Mini Kit (5 PRIME Inc.) and nucleotide sequences determined at Macrogen (Seoul, Korea). Primer pairs (see Table S6) were designed using Primer3 software^41^, and amplifications were carried out using a *Taq* DNA polymerase (Biotaq, Bioline Ltd., London) or a high-fidelity mix (PrimeSTAR HS, Takara). When necessary for site directed mutagenesis, we used the Sm^r^/Sp^r^ cassette from pHP45Ω or the Gm^r^ cassette from pJQ200SK amplified by PCR with specific primers that contained the desired terminal restriction sites for disruption cloning (Table S6).

For functional analyses, the *gin* genes in *Pst* DSM 4166 and in pGInt0 were subjected to site-directed mutagenesis. Non-polar mutations were introduced by filling-in unique restriction sites internal to the CDSs or by digestion with enzymes cutting twice in the CDS followed by religation, with the concomitant change in the reading frame. Enzymes used for mutagenesis of *Pst* DSM 4166 genes were: *ginA*, AccI; *ginB*, SacI; *ginC*, EcoRI; for pGInt0: *ginA*, NcoI, resulting in the deletion of 657 nt; *ginB*, AvrII; *ginC*, SexAI. Gene *ginD* was disrupted by insertion of the Gm^r^ cassette, in strain DSM 4166, or with the Sm^r^/Sp^r^ cassette, in pGInt0, in the single StuI site occurring in this gene. When necessary, mutant DNA was exchanged into strain *Pst* DSM 4166 using pK18mobsacB. Complementation of mutants was done *in trans* using individual *gin* genes amplified with specifically designed primers (Table S6), cloned under control of the *P*_BAD_ promoter into pJN105. These clones were then transferred to the corresponding mutant and GInt excision examined in culture medium containing 0.5% arabinose, essentially as described^42^. Strain UPN828 (*ginD*::Gm^r^) was complemented with a wt copy of *ginD* inserted alongside the mutant copy.

### Analysis of the circularization and transfer of GInts

We followed a nested PCR procedure to evaluate excision and circularization of GInts and pGInt0, but using primers specific for each GInt because of sequence divergence. We used specific outward primers annealing close to the putative ends of each element to test for circularization, and specific primers outside of the element, and directed inwards, to amplify the scar left after excision (see Fig. 1 and Table S6). To test this in axenic cultures, 100 μl samples of KB cultures growing at 28 °C with shaking were taken after 6, 24, 48, 72 or 98 hours of incubation, mixed with 400 μl of sterile distilled water, boiled for 10 minutes and centrifuged. To evaluate circularization of the Pht-PAI *in planta*, a disc from an inoculated bean leaf or pod was cut with a n° 4 cork borer at 1, 2, 3, 4 and 7 days post-inoculation and was homogenized in sterile ¼ strength Ringers solution, before purifying the DNA for PCR as described by Llop *et al*.^43^. The first PCR round was carried out in a final volume of 12.5 μl with 2.5 μl of cell lysates from liquid cultures or 0.5 μl of purified DNA from plants; 2.5 μl of the resulting PCR was used as template for a 25 μl second round nested PCR. Experiments were repeated at least three independent times. Five to twenty independently cloned PCR products were sequenced for each combination of type of amplicon and strain.

To evaluate the transfer of GInts from *P. syringae* pv. phaseolicola 1448A and *Pst* DSM 4166 to two strains of *P. syringae* pv. syringae, we constructed derivatives containing an antibiotic resistance cassette within their cargo DNA. UPN779 derives from strain 1448A after the Sm^r^/Sp^r^ cassette was inserted in the NruI site immediately 3′ of PSPPH_4320. UPN816 derives from DSM 4166 and contains the Sm^r^/Sp^r^ cassette replacing a 450 nt HincII fragment internal to PSTAA_0898, a type I restriction-modification system subunit R. We co-inoculated in KB, as donor:receptor, strains UPN779:B728a and UPN816:UPN853. After overnight growth at 25 °C with shaking, we plated serial dilutions of the UPN779:B728a culture on MG + Cu + Sp and MG + Sm + Sp, to select for B728a clones that had acquired the Pht-PAI, and of the UPN816:UPN853 culture on KB + Sp + Km, to select for UPN853 that had acquired the GInt. Since both strains UPN779 and B728a are pathogens of bean, we also tested transmission *in planta*. Inoculations and incubation of plant material were carried out essentially as described^44^. For co-inoculation, equal volumes of cell suspensions of strains UPN779 and B728a adjusted to an OD_600_ of 0.1 (approximately 5 × 10^7^ colony-forming units mL^−1^) were mixed and injected subepidermically on fresh bean pods (cv. Helda), which were deposited over moist filter paper in a plastic box with a hermetic lid. After incubation at room temperature, disks were removed from the inoculated area after 1, 2 and 4 days post-inoculation using a cork borer (no. 4), thoroughly homogenized as above and serial dilutions were plated onto selective media. Experiments to evaluate transfer of GInts were repeated at least six independent times.

### Construction and transfer of pGInt0

pGInt0 is an artificial construction mimicking the putative circular intermediate of the GInt from *Pst* DSM 4166, except that the cargo DNA has been replaced by the cloning vector pUC57-Kan (accession no. JF826242), which cannot replicate in *Pseudomonas*. It was synthesized by GeneScript (Piscataway, NJ, USA) and it consists of the last 481 nt from the 3′ end of the GInt followed by the first 5,802 nt of the 5′ end (coordinates 983899–984379 joined to 960530–966331, from accession no. CP002622), cloned in the polylinker of pUC57-Kan in the orientation opposite to the P_*lac*_ promoter. Except for the relative orientation of the *gin* operon and the 3′ end, and a nucleotide change to eliminate an EcoRI site internal to *ginC* made to facilitate further manipulations, the sequence of the insert from pGInt0 is identical to that of strain DSM 4166.

Integration of pGInt0 in *Pseudomonas* was analysed by electroporating plasmid DNA purified from *E. coli* by alkaline lysis: since pGInt0 is cloned in a suicide vector for *Pseudomonas*, the Km^r^ clones resulting from transformation must contain an integrated copy of the artificial GInt. Integration into the canonical *attB* was analysed by PCR (Table S6).

### Reverse transcription-PCR analysis

DNA-free RNA was obtained from bacterial cultures grown overnight in KB using TriPure Isolation Reagent (Roche Diagnostics) and Ambion TURBO DNA-free Kit (Life Technologies). The concentration and purity of RNA was determined spectrophotometrically, and its integrity confirmed by electrophoresis in agarose gels. cDNA was synthesized from RNA using the ImProm-II reverse transcriptase system (Promega), following the manufacturer’s recommendations. Both random hexanucleotides (Promega) and primers specific for sense and antisense transcripts in the *gin* operon were used for the reaction (Table S6). Reverse transcription was carried out at 25 °C for 5 min for primer annealing, 60 minutes at 42 °C for reverse transcription, and 15 minutes at 70 °C for enzyme inactivation. PCR amplification was carried for 30 cycles (94 °C for 30 s, 55 °C for 30 s and 72 °C for 30 s) plus a final extension step of 6 min at 72 °C. Control reactions included PCR amplification of RNA subjected to a reverse transcription reaction lacking reverse transcriptase, to verify the absence of contaminating DNA; amplification of purified DNA, to verify the reaction conditions, and amplification of an internal fragment of gene *gyrB*, to confirm the synthesis of cDNA.

### Bioinformatics

Partial or complete sequences of *rpoD, gyrB, acnB, gap1, gltA* and *gin* genes were obtained directly from the NCBI databases. Sequences of *rpoD, gyrB, acnB, gap1* and *gltA* were concatenated and treated as a single sequence containing 6,283 nt. Multiple-sequence alignments using Muscle, determination of the optimal nucleotide substitution model and phylogenetic tree construction were done using MEGA6^45^. Trees were constructed with Maximum likelihood methods, using the General Time Reversible model, and confidence levels of the branching points were determined using 500 bootstraps replicates. Searches for sequence similarity against the NCBI databases were done using the BLAST algorithms^46^. Alignments were done using the Needle and T-Coffee tools at the EMBL site (http://www.ebi.ac.uk/services). Sequence logos were created with WebLogo 3^47^. Genome and nucleotide sequences were visualized and manipulated using the Artemis genome browser and Blast comparisons using ACT^48^. Promoters were predicted using the online BPROM server (http://www.softberry.com)^49^. Prediction of protein structure was done using the Phyre2 web server (http://www.sbg.bio.ic.ac.uk/phyre2)^50^. To search for homologues of the *ginABCD* operon, we proceed to an extensive and very permissive *in silico* survey within complete prokaryotic genomes. We considered that a genome harbours a *gin* operon homolog if it contains, using BlastP with an E-value cut-off threshold of 0.1, three consecutive CDSs whose products are homologous to GinA, GinB and GinC; we excluded GinD from the search because of its small size and because this CDS is not always annotated.

## Additional Information

**Accession codes**: Sequences of the artificial GInt pGInt0 and of the GInt from strain P. syringae pv. phaseolicola CYL314 are deposited in the EMBL under accession numbers LT671993 and LT671994, respectively.

**How to cite this article:** Bardaji, L. *et al*. Four genes essential for recombination define GInts, a new type of mobile genomic island widespread in bacteria. *Sci. Rep.* **7**, 46254; doi: 10.1038/srep46254 (2017).

**Publisher's note:** Springer Nature remains neutral with regard to jurisdictional claims in published maps and institutional affiliations.

## Supplementary Material

Supplementary Information
