# Supplementary material for: Four genes essential for recombination define GInts, a new type of mobile genomic island widespread in bacteria
Source: Sci Rep. 2017 Apr 10;7:46254. doi: 10.1038/srep46254 (PMC5385486; doi:10.1038/srep46254)
Supplement: Supplementary Information [file srep46254-s1.pdf]

SUPPLEMENTARY FIGURES AND TABLES

Four genes essential for recombination define GInts, a new type of mobile genomic island widespread in bacteria

Leire Bardaji,[1] Myriam Echeverría,[1] Pablo Rodríguez-Palenzuela,[2] Pedro M. Martínez-García,[2,3] Jesús Murillo[1*]

[1] Departamento de Producción Agraria, Escuela Técnica Superior de Ingenieros Agrónomos, Universidad Pública de Navarra, 31006 Pamplona, Spain

[2] Centro de Biotecnología y Genómica de Plantas, E.T.S. Ingenieros Agrónomos, Universidad Politécnica de Madrid, Campus de Montegancedo, E-28223 Pozuelo de Alarcón, Madrid, Spain

[3] Instituto de Hortofruticultura Subtropical y Mediterránea "La Mayora", Universidad de Málaga-Consejo Superior de Investigaciones Científicas (IHSM-UMA-CSIC), Área de Genética, Facultad de Ciencias, Campus Teatinos s/n, 29010 Málaga, Spain

***For correspondence**: Jesús Murillo, E-mail: jesus.murillo@unavarra.es

Supplementary Figure S1. Conservation of key amino acid residues in GinA, GinB and GinC proteins and their mutated sequences.

Supplementary Figure S2. GInts are exchanged horizontally among pseudomonads.

Supplementary Figure S3. GInts acquire the cargo DNA mainly from closely related bacteria.

Supplementary Table S1. Characteristics of representative GInts highly related to that from *Ps* pv. phaseolicola 1448A (Pht-PAI).

Supplementary Table S2. Circularisation of GInts and sequence of the resulting *attB* and *attI* sites in selected strains.

Supplementary Table S3. Examples of GInts found in different taxonomical groups of bacteria.

Supplementary Table S4. Examples of significant clusters of genes found in the cargo DNA of diverse GInts.

Supplementary Table S5. Bacterial strains and plasmids used in this study.

Supplementary Table S6. Primers used in this study.

```
        181                                                        240
GinA    RTRLLEIMHPDSAENPFSDEAIRLRNYIILLLGIDMGLRRSEMLLIKTSDIHWHSRQLAV
GinA'   RTRLLEIMHPDSAENPFSDEAIRLRNYIILLLGIDMGLRRSEMLLIKTSDIHWHSRQLAV

        241                                                        300
GinA    VNLEDESLDPRTMAPQFKTHERMLVMTDDLYDAITEYESKYRHRKPRSGTSQARRHPFLL
GinA'   VNLEDESLDPRTMAPQFKTHERMLVMTDDLYDAITEYESKYRHRKPRSGTSQARRHPFLL

        301                                                        360
GinA    VAHKRNEGGPLTIKAVDGVLSRVREIAPELAHVHTHILRHDAVYTML-ESMREELAALTP
GinA'   VAHKRNEGGPLTIKAVDGVLSRVREIAPELAHVHTHILRHDAVSTRCWKACVRNWQRLHR

        361                                                        420
GinA    EDRTTQIQKTLTWMFGWSPDSNMPGHYGAKFWKEEADKAIQKR-AKRFTAIRQKAGTTQG
GinA'   RIAPLKFKRHSHGCSAGVPTRTCPATTARNSGRKRQTRRYRKGPSDSRPSVRRPARHKEV
```

```
        181                                                        240
GinB    IHEQFCHGHLGFEQYLYLRMLMIYGQRGTQVRMMVFGDFTKGDQGCKVRFHWAKQNDDEA
GinB'   IHEQFCHGHLGFEQYLYLRMLMIYGQRGTQVRMMVFGDFTKGDQGCKVRFHWAKQNDDEA

        241                                                        300
GinB    GWRAKAETFSLDEGLYNTVQAYKAMVLAQLWQTYPDGADWNAAIENVPVFRKKLDGETGA
GinB'   GWRAKAETFSLDEGLYNTVQAYKAMVLAQLWQTYPDGADWNAAIENVPVFRKKLDGETGG

        301                                                        360
GinB    RDRVNPPVLLDTPLQKALEDAPQPTFHIGSSTIKRWLECIERMKGFPVSPRTHQPLKVTR
GinB'   IG*

        361                                                        420
GinB    GHRFRHTLGTDLSNAGLDEWTMARALMHKNTQAVRKYRAVSPELLALIDAKMSDHLALVV
```

```
        61                                                         120
GinC    ALGSTKAAPKAMQAPFTEFAKAILVYRRVYLQKKAMTDWLRAMIALEFALFELTGTRDVT
GinC'   ALGSTKAAPKAMQAPFTEFAKAILVYRRVYLQKKAMTDWLRAMIALELIRPVRVDRHAGC

        121                                                        180
GinC    RVSAAVCNKACEHLNRHWTKGNTAYQHGLALEALITLMRAKKLLKSDFRWTSPLTLMPLG
GinC'   DAGVRRCL*

        481                                                        540
GinC    NLKKTSLFARYGYPGVKVNTHAFRHELNTRMHQAGLSQLLIDAFSGRTTRGSVYNHETIE
```

Supplementary Figure S1. Conservation of key amino acid residues in GinA, GinB and GinC proteins and their mutated sequences. Pairwise alignment of GinA, GinB and GinC proteins from *P. stutzeri* DSM4166 with the deduced products of the corresponding mutant genes (from strains UPN821, UPN823, and UPN825), indicated with an apostrophe; for clarity, only part of the alignments are shown. Amino acid residues before the out-of-frame mutations appear in red. Residues boxed in grey correspond to those conserved in at least 90 % of the homologues from the Psi-Blast analysis carried out by Phyre2 (1000 sequences were aligned). Numbers indicate amino acid positions. An asterisk indicate the end of the protein.

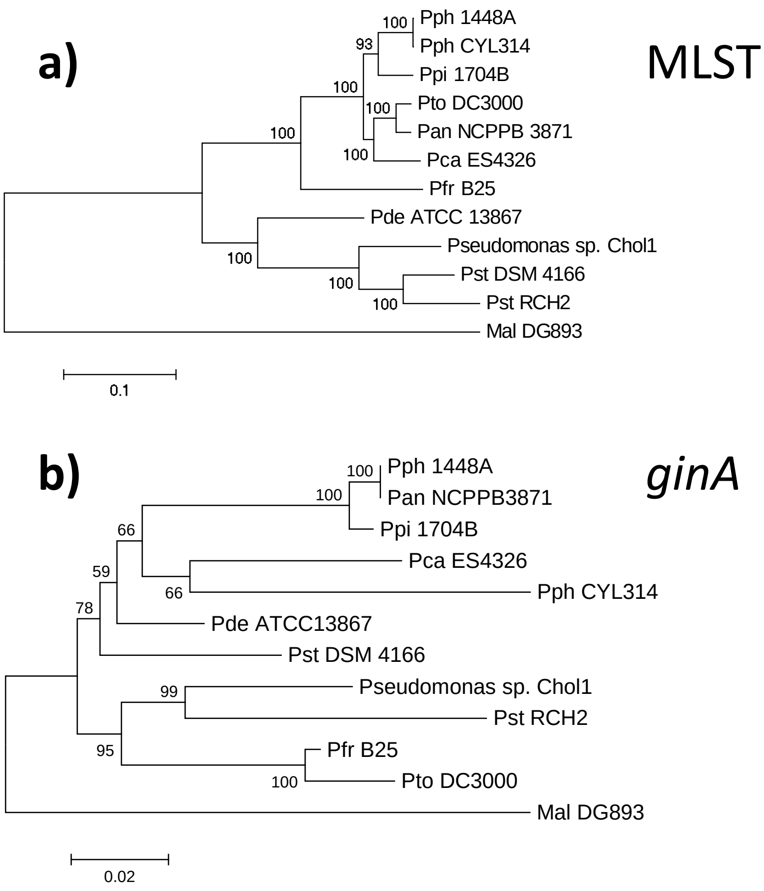

Supplementary Figure S2. GInts are exchanged horizontally among pseudomonads. Phylogenetic analyses were done with selected GInts whose *gin* operons showed an overall nucleotide identity higher than 70 % to that from the Pht-PAI of *P. syringae* pv. phaseolicola 1448A (see Table S1). Maximum likelihood trees were constructed with concatenated partial or complete sequences of *rpoD*, *gyrB*, *acnB*, *gap1* and *gltA* **(a)** and the complete nucleotide sequence of *ginA* **(b)**, based on the General Time Reversible model. Trees constructed separately using the sequences from *ginB*, *ginC*, and *ginD* showed a topology similar to that of *ginA* and are not shown for simplicity. Trees are drawn to scale, with branch lengths measured in the number of substitutions per site. Numbers in branches indicate per cent bootstrap values with 500 replicates. Abbreviations: Mal, *Marinobacter algicola*; Pan, *Pseudomonas syringae* pv. actinidiae; Pca, *P. cannabina* pv. alisalensis; Pde, *P. denitrificans*; Pfr, *P. fragi*; Pph, *P. syringae* pv. phaseolicola; Ppi, *P. syringae* pv. pisi; Pst, *P. stutzeri*; Pto, *P. syringae* pv. tomato.

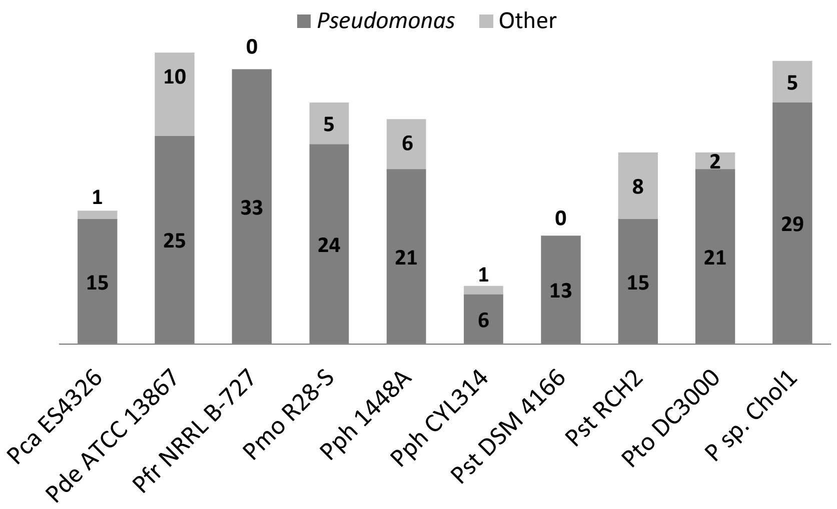

Supplementary Figure S3. GInts acquire the cargo DNA mainly from closely related bacteria. The CDSs from the cargo DNA of GInts from strains of *Pseudomonas* (Table S1) was analysed using blastp. Bars are proportional to the number of proteins whose closest homologue was found in other *Pseudomonas* species (dark grey) or in bacteria from other genera (light grey), with total number of proteins for each category shown within or above bars. Abbreviations: Pca, *Pseudomonas cannabina* pv. alisalensis; Pde, *P. denitrificans*; Pfr, *P. fragi*; Pmo, *P. moraviensis*; Pph, *P. syringae* pv. phaseolicola; Pst, *P. stutzeri*; Pto, *P. syringae* pv. tomato. P sp., *Pseudomonas* sp*.* Chol1.

Supplementary Table S1. Characteristics of representative GInts highly related to that from *Ps* pv. phaseolicola 1448A (Pht-PAI).[a]

Species	Strain	Acc. no.	*gin* operon	Cargo DNA	Size (kb)	% id.[b]	Status[c]
Pseudomonas syringae							
pv. phaseolicola[d]	1448A	CP000058	PSPPH_4294/ PSPPH_4297	PSPPH_4298/ PSPPH_4324	38.0	-	complete
	CYL314	LT671994	CYL314_016/ CYL314_019	CYL314_020/ CYL314_028	15.6	70.7	complete
pv. tomato	DC3000	AE016853	PSPTO_4603/ PSPTO_4606	PSPTO_4607/ PSPTO_4630	34.8	86.7	Complete
pv. pisi	1704B	GL384900	PSYPI_04159/ PSYPI_04144	PSYPI_04139/ unk[e]	unk	97.5	Incomplete
***Pseudomonas* sp.**							
	Chol1	AMSL01000014	C211_02301/ C211_02316	C211_02321/ C211_02486	43.5	86.0	complete
***P. cannabina* pv. alisalensis**							
	ES4326	NZ_GL385049	PMA4326_RS23690/ PMA4326_RS23705	PMA4326_RS23710/ PMA4326_RS23775	≥18.9	88.0	incomplete
P. chloritidismutans							
	AW-1	AOFQ01000065	F753_21675/ F753_21690	F753_21695/ F753_21815	≥31.8	86.9	incomplete
P. denitrificans							
	ATCC 13867	CP004143	H681_04555 / H681_04540	H681_04535 / H681_04370	47.8	88.2	complete
P. fragi							
	B25	NZ_JH604622	O5A_RS0101405/ O5A_RS0101390	O5A_RS0101385/ O5A_RS0101225	≥34.9	86.0	incomplete
	NRRL B-727	LT629783	SAMN05216594_3852/ SAMN05216594_3849	SAMN05216594_3848/ SAMN05216594_3815	35.2	86.1	complete
P. moraviensis							
	R28-S	CM002330	PMO01_23370/ PMO01_23385	PMO01_23390/ PMO01_23530	35.6	63.7	complete
P. stutzeri							
	DSM 4166	CP002622	PSTAA_0885/ PSTAA_0887	PSTAA_0888/ PSTAA_0900	23.8	87.0	complete

	RCH2	CP003071	Psest_3432/ Psest_3429	Psest_3428/ Psest_3405	29.2	86.0	complete
Hahella ganghwensis							
	DSM 17046	AQXX01000141	F566_27925/ F566_27910	F566_27905/ F566_27860	17.3	63.9	complete
Marinobacter algicola							
	DG893	ABCP01000041	MDG893_13179/ MDG893_13164	MDG893_13159/ MDG893_13054	≥27	79.0	incomplete
Teredinibacter turnereae							
	T7902	NZ_KB900633	YUK_04735/ YUK_04750	YUK_04755/ YUK_04930	52.6	51.7	complete

[a] We include here GInts whose entire *gin* operon shows nucleotide identities higher than 50 % with that of *P. syringae* pv. phaseolicola 1448A. The *gin* operon from strain CYL314 contains a large internal deletion affecting *ginB* and *ginC* and resulting in a lower identity score.

[b] Percentage of nucleotide identity between the *gin* operon from this element and that from *P. syringae* pv. phaseolicola 1448A in pairwise global comparisons using the Needle tool from EMBOSS (Needleman-Wunsch algorithm; http://www.ebi.ac.uk/Tools/emboss/).

[c] Complete indicates that we were able to identify a continuous assembly including the 3' end of the GInt, which was generally detected by genome comparisons with closely related bacteria. Incomplete GInts are the result of incomplete assemblies or difficulties in unambiguously identifying the poorly conserved 3' end.

[d] The Pht-PAI is present as a nearly identical copy in all strains of *P. syringae* pv. phaseolicola and *P. syringae* pv. actinidiae that produce phaseolotoxin. The genomes of these strains are not mentioned here for clarity.

[e] unk, unknown

Supplementary Table S2. Circularisation of GInts and sequence of the resulting *attB* and *attI* sites in selected strains.

Strains	*attI* / *attB*[a]	*attL* and *attR* of GInts[b]	Reconstructed *attI*[c]	Reconstructed *attB*[c]
Pseudomonas syringae				
pv. phaseolicola 1448A	YES / NO	L tgtagaCGTA-AGAGCCtgt<br>R gatatcCGTATTGAGCCatg	gatagaCGTAAGAGCCtgt	
pv. pisi 1704B	YES / YES	L tgtagaCGTA-AGAGCCtgt<br>R gatatcCGTATTGAGCCatg	gatagaCGTAAGAGCCtgt	tgtatcCGTATTGAGCCatg or<br>tgt--CCGTATTGAGCCatg or<br>tgt------ATTGAGCCatg
pv. tomato DC3000	YES / YES	L tgtagaCGTA-AGAGCCtgt<br>R gataccGATATTGAGCCatg	gatagaCGTAAGAGCCtgt	tgtagaCGTATTGAGCCatg
P. cannabina pv. alisalensis ES4326	YES / YES	L tgtagaCGTA-AGAGCCtgt<br>R gatggcTCTATTGAGCCatg	gatagaCGTAAGAGCCtgt	tgtggcTCTATTGAGCCatg
P. fragi B25	YES / NO	L tgaaaaCGTA-AGAGCCtgt<br>R gataagCGTATTGAGCCatg	gataaaCGTAAGAGCCtgt	
P. stutzeri DSM4166	YES / NO	L tgtagaCGTA-AGAGCCggt<br>R gattgaTGAATTGAGCCatg	gatagaCGTAAGAGCCggt or<br>gattgaTGAAAGAGCCggt	
P. putida KT2440::pGInt0	YES / YES	L tgtagaCGTA-AGAGCCggt<br>R gattgaTGAATTGAGCCaaa	gatagaCGTAAGAGCCggt or<br>gattgaTGAAAGAGCCggt	tgtagaCGTATTGAGCCaaa

[a] YES and NO indicate whether or not the circular intermediate (*attI*) and the scar (*attB*) were detected by PCR.
[b] L and R indicate the putative left and right borders of the GInt, respectively. Dashes indicate gaps introduced to maximize the alignment
[c] Sequences shaded in orange and grey originate from the GInt and the bacterium, respectively. Dashes indicate gaps introduced to maximize the alignment.

Supplementary Table S3. Examples of GInts found in different taxonomical groups of bacteria.[a]

Class / Order/ Strain	Accession no.	Inserted in gene/product[b]	*ginA*, Locus_tag	Size of GInt (kb)	*attL-attR*[C]
**PHYLUM Bacteroidetes**					
**Flavobacteria/ Flavobacteriales/**					
Chryseobacterium artocarpi UTM-3	NZ_MAYH01000023.1	*ssb*	BBI01_07255	ND	ND
**PHYLUM Firmicutes**					
**Clostridia/ Clostridiales/**					
Roseburia inulinivorans DSM 16841	ACFY01000093	ATP-dependent DNA helicase	ROSEINA2194_ 02362	ND	ND
**Erysipelotrichia/ Erysipelotrichales/**					
Eubacterium cylindroides T2-87	NC_021019	Rad3-related DNA helicase	EC1_RS04255	ND	ND
**PHYLUM Verrucomicrobia**					
**Opitutae/ Opitutales/**					
Opitutus terrae PB90	CP001032	*ssb*	Oter_2289	9.7	L:GTGCCGTTCT<br>R:GTGCCGTTCT
**PHYLUM Proteobacteria**					
**Alphaproteobacteria/ Rhizobiales/**					
Methylobacterium populi BJ001	CP001029	O-acyl transferase	Mpop_3234	8.3	L:GCCGGAAAACGGAT<br>R:GCCGGAGAACGGAT
**Betaproteobacteria/ Burkholderiales/**					
Ralstonia solanacearum PSI07	FP885906	*ssb*	RPSI07_2980	35.0	L:CCGTTCTAAT<br>R:CCGTTTTGAAT

**Gammaproteobacteria/ Alteromonadales/**					
Shewanella baltica BA175	CP002767	*ssb*	Sbal175_0650	75.8	L:CCGTTTTAATT<br>R:CCTTTTTAATT
**Gammaproteobacteria/ Pseudomonadales/**					
Pseudomonas aeruginosa PA_D16	CP012581	ribonucleoside-diphosphate reductase subunit alpha	GInt_1; A6695_20385	40.3	L:TGCCAGTGAGCCCG<br>R:TGCCAGTGATCGCG
			GInt_2; A6695_21830	35.3	L:TGCCAGTGAGCCC<br>R:TGCCA-TGATCGC
P. syringae pv. syringae UMAF0158	CP005970	*ssb*	PSYRMG_13425	19.7	
P. syringae pv. myricae AZ84488	LGLA0100042	*ssb*	AC510_0694	30.7	
**Gammaproteobacteria/ Vibrionales/**					
Vibrio parahaemolyticus RIMD 2210633	BA000031	DNA-binding protein HU-2	VP2910	15.7	L:TGATTCTTTCAG<br>R:TGCTTCTTTCAG

[a] In all cases the *gin* operon was found to be associated to region of cargo DNA, of variable size, whose presence was deduced by comparison with the genome of the closest relative without GInt. Sizes of GInts are in kb. ND: not determined; the ends of the GInt could not be determined because its DNA is not assembled into a continuous contig, because the ends are degenerate, with deletions or insertions of other mobile elements, or because the ends could not be accurately defined by comparison with an appropriate genome lacking the GInt.

[b] All *ssb* genes are homologous.

[c] Deduced Left (L) and right (R) direct imperfect repeats bordering the GInts, but not confirmed experimentally.

Supplementary Table S4. Examples of significant clusters of genes found in the cargo DNA of diverse GInts.

				**Homologue**	
**Strain**	**Number of genes**	**Gene cluster**	**Possible function or products**	**Syntenic region**	**Organism**
Pseudomonas sp. Chol1					
	6	C211_02326/ C211_02351	Biofilm formation	PSTAA_2727/ PSTAA_2720	*P. stutzeri* DSM 4166
	9	C211_02391/ C211_02431	Mercury resistance	PSPA7_0089/ PSPA7_0097	*P. aeruginosa* PA7
	6	C211_02461/ C211_02486	DNA restriction and modification	PSTAA_0888/ PSTAA_0894	*P. stutzeri* DSM 4166
P. aeruginosa PA_D16					
	7	A6695_20500/ A6695_20530	Tetracycline and aminoglycoside resistance	coordinates 28414 to 33695 accession nº KM649682	*Stenotrophomonas maltophilia* GZP-Sm1, Sm1-MDRGI genomic island
	3	A6695_20495/ A6695_20485	Chloramphenicol and aminoglycoside resistance	ETN48_p0093/ ETN48_p0095	*Escherichia coli* O102-ST405 plasmid pETN48
	5	A6695_20475/ A6695_20455	Trimethoprim and quaternary compounds resistance	AM278_28040/ AM278_28060	*Klebsiella pneumoniae* UCLAOXA232KP plasmid
P. denitrificans ATCC 13867					
	6	H681_04460/ H681_04435	Transporter systems	PP_3954/ PP_3959	*P. putida* KT2440
P. fragi NRRL B-727					
	8	SAMN05216594_3835/ SAMN05216594_3842	Copper resistance	PVE_R1G6098/ PVE_R1G6092	*P. veronii* 1YdBTEX2
P. moraviensis R28-S					
	6	PMO01_23485/ PMO01_23510	Phosphite transport	PA2G_01873/ PA2G_01878	*P. aeruginosa* 2192
P. stutzeri DSM 4166					
	7	PSTAA_0888/ PSTAA_0894	Nickel transport	Psest_0941/ Psest_0936	*P. stutzeri* RCH2
Pseudomonas syringae					

pv. phaseolicola 1448A				
22	PSPPH_4299/ PSPPH_4319	Phaseolotoxin production	-	*P. syringae* pv. syringae CFBP3388
pv. tomato DC3000				
17	PSPTO_4608/ PSPTO_4624	Bacterial fittness	Psyr_1475/ Psyr_1491	*P. syringae* pv. syringae B728a
Shewanella baltica BA175				
4	Sbal175_0670/ Sbal175_0673	Insecticidal toxin complex	Sbal678_0614/ Sbal678_0617	*Shewanella baltica OS678*
Teredinibacter turnerae T7902				
3	YUK_04845/ YUK_04855	Carbohydrate transport	F566_08265/ F566_08275	*H. ganghwensis* DSM 17046
3	YUK_04920/ YUK_04930	DNA restriction and modification	B013_19835/ B013_19825	*T. turnerae* T0609

Supplementary Table S5. Bacterial strains and plasmids used in this study.[a]

Strain or plasmid	Host or place of isolation; description; synonyms	Source or reference
**Bacteria**		
Escherichia coli		
NEB10-beta	Host for genetic manipulations.	New England Biolabs
Pseudomonas cannabina		
pv. alisalensis ES4326	*Raphanus sativus*; pathogen of crucifers; CFBP 1637, NCPPB 1820	CFBP
P. fluorescens SBW25	*Beta vulgaris*; plant growth-promoting rhizobacterium.	[1]
P. fragi B25	Type strain; synonyms are NRRL B-25, CFBP 4556, ATCC 4973	CFBP
P. putida KT2440		M. Espinosa
P. stutzeri		
DSM 4166[b]	Rhizosphere of *Sorghum* mutans; Sp^r.	DSMZ
UPN816[b]	DSM 4166 containing an Sm^r/Sp^r cassette inserted in the GInt, within PSTAA_0898, to evaluate the transfer of the GInt.	This work
UPN821	DSM 4166 with an AccI site in *ginA* filled-in, resulting in a frameshift mutation changing the 76 C-terminal amino acids.	This work
UPN823	DSM 4166 with the unique SacI site in *ginB* blunted, resulting in a premature stop codon in residue 300.	This work
UPN825	DSM 4166 with the unique EcoRI site in *ginC* filled-in, resulting in a premature stop codon in residue 129.	This work
UPN828	DSM 4166 with *ginD* disrupted by insertion of the Gm^r cassette in the unique StuI site.	This work
P. syringae		
pv. phaseolicola		
CYL314	*Phaseolus vulgaris*; pathogen of bean.	[2]
1448A	*Phaseolus vulgaris*; pathogen of bean.	[3]
UPN779	Strain 1448A with a Sm^r/Sp^r cassette inserted in the NruI site immediately after PSPPH_4320 in the Pht-PAI.	This work
pv. pisi 1704B	*Pisum sativum*; pathogen of pea; CFBP 2709.	CFBP
pv. syringae		
B728a	*Phaseolus vulgaris*; pathogen of bean, Cu^r, Rif^r, Sm^r.	G.W. Sundin[4]
UMAF0158	*Mangifera indica*; pathogen of mango and tomato.	[5]

UPN853	UMAF0158 with a chromosomal insertion of IS-Ω-*Km/hah*.	This work
pv. tomato DC3000	*Solanum lycopersicon*; pathogen of tomato	[6,7]
**Plasmids**		
pGInt0	Artificial construct containing the GInt from *P. stutzeri* DSM 4166, without the cargo DNA and in closed conformation, cloned in pUC57, Km^r.	This work
pGInt0.1	Plasmid recovered from *P. putida* KT2440::pGInt0; it is pGInt0, but contains three nt changes in its *attI*, making it more efficient for integration into diverse pseudomonads.	This work
pGInt0A	pGInt0 digested with NcoI enzyme and religated resulting in a deletion of 657 nucleotides in *ginA*.	This work
pGInt0B	pGInt0 with the unique AvrII site filled-in, resulting in a frame shift mutation in *ginB*.	This work
pGInt0C	pGInt0 with the unique SexAI site filled-in, resulting in a frame shift mutation in *ginC*.	This work
pGInt0D	pGInt0 with the Gm^r cassette from pJQ200SK inserted in the unique StuI site of *ginD*.	This work
pHP45Ω	Source of the Sm^r/Sp^r cassette.	[8]
pJN105	*araC*-P_{BAD} cassette cloned in pBBR1MCS-5.	[9]
pJN105::*ginA*	*ginA* cloned in pJN105.	This work
pJN105::*ginB*	*ginB* cloned in pJN105.	This work
pJN105::*ginC*	*ginC* cloned in pJN105.	This work
pJN106	P_{BAD}::*lacZ* derivative of pJN105, Km^r.	[9]
pJQ200SK	Source of the Gm^r cassette.	[10]
pK18mobsacB	Suicide mobilizable vector for marker-exchange mutagenesis in *Pseudomonas*; *sacB*, *oriT*, Km^r.	[11]
pSCR001	IS-Ω-*Km/hah* plasmid, Mob^+, Km^r.	[12]

[a] CFBP, Collection Française de Bactéries Phytopathogènes, France; Cu^r, copper resistance; DSMZ, German Collection of Microorganisms and Cell Cultures, Germany; Gm^r, gentamicin resistance; Km^r, kanamycin resistance; NCPPB, National Collection of Plant Pathogenic Bacteria, UK; Rif^r, rifampicin resistance; Sm^r, streptomycin resistance; Sp^r, spectinomycin resistance.

[b] *P. stutzeri* DSM 4166 is resistant to no more than 50 μg spectinomycin/ml, whereas UPN816 is resistant to 200 μg spectinomycin/ml.

Supplementary Table S6. Primers used in this study.

From	Primer name	Sequence	Fragment[a]	Ref[b]
pJQ200SK				
	Gm_pJQ200_F	GCAAAAGAAAATGCCGATG	Gm^R cassette	
	Gm_pJQ200_R	CCTTGCCGTAGAAGAACAGC		
Pfu SBW25				
	GIntO_R_SBw	CACCACAGCAGGTAGGAAGTC	with fasout_stz_4, amplification of the insertion of pGInt0	
	Fasbor_LL3_SB	CAGGTAGCCGATGTTCAGGT	with GIntO_R_SBw amplification of *attB*	
Ppu KT2440				
	GIntO_R_put	GAACGCAATGACGACGGATG	with fasout_stz_4, amplification of the insertion of pGInt0	
	Fasbor_RR3_put	CGGATTCTTCAGGCTCTGTG	with Ptz-RNA-F1 amplification of *attB*	
Pph 1448A				
	fas_intClon_F[c]	ACTAGTCTCACCTATGGCAACCCAAT	Pht-PAI	
	fas_intClon_R	TTGATGTCCAGTTCGACCAA		
	fasout_1	ATGTCCAAGGTTTGCTTGCT	Round 1 *attI*	
	fasout_2	TAGCTGAAGGTTCTGCGTGA		
	fasout_3	GCTGTTCGAGGTGAGAAATG	Round 2 *attI*	
	fasout_4	CTGGTACGAACTGACCATCG		
	fasbor_LL2	ATTCTTGCGACTGCATTTCT	*attB*	13
	fasbor_RR	CTGTCACCGACAATGTCACC	Round 1 *attB* with fasbor_LL2	13
	fasbor_RR2	TGTTCCTGACTGCTGGACTG	Round 2 *attB* with fasbor_LL2	13
Ppi 1704B				
	fasout_pisi_2	ACCCAAAATCAGCAAAAGC	*attI* with fasout_1	
	fasout_pisi_4	AAGATGCTGTACAGGCTCGAAAG	*attI* with fasout_3	
	FAS23F_pisi	TCAGAAGACTCCGTGCTCG	Internal fragment of cargo DNA	
	FAS25F2	TCAAGGCGGAAAAGAGTGTC		
	FAS27F	TGGTCATGGACACTCTTTTCC	Internal fragment of cargo DNA	
	FAS27R	GCACTGGATTACCTCCAAGG		
Pto DC3000				
	fasout_To_1	GCTCAAGCACAATAAGATGCTC	Round 1 *attI*	
	fasout_To_2	CAATCAAGTAAACCCATTCCAC		
	fasout_To_3	ACGAGCACCATTCACAATCTG	Round 2 *attI*	
	fasout_To_4	TTCGGCGGAGAGTGAGATTAC		
Pph CYL314				
	fasout_CYL_1	TGAGGGCGCTGTTCTAGATG	Round 1 *attI*	
	fasout_CYL_2	GGTTAGCCAGTCCACTCTTG		
	fasout_CYL_3	AACACAGCCAGATGCTCAAG	Round 2 *attI*	
	fasout_CYL_4	AATGCGGGGACTGAAACATC		
Pfr B25				
	fasout_frg_1	GCTGTTCGAGGCGAGAAATC	Round 1 *attI*	
	fasout_frg_2	GACAGCGCCTTCATCAAACC		
	fasout_frg_3	AGCAGCTTTCTACGAGCACC	Round 2 *attI*	

fasout_frg_4	ATAGGCGAGCACATTCAAGC	
fasbor_frg_1	TCCACAGGCAGCAGATTGAG	Round 1 *attB*
fasbor_frg_2	TCGGCGTAAACCTGATCCAG	
fasbor_frg_3	GTAATTGATGCTGGCCTGGC	Round 2 *attB*
farbor_frg_4	TCTTCCACCACTTCGCGTAC	
Pca ES4326		
fasbor_mac_R2	CTGCATTCGGGTAGCTTTGC	Round 1 *attB* with fasbor_LL2
fasbor_mac_R4	CTGGAGTTATTCAAGGCGCG	Round 2 *attB* with fasbor_LL2
Mac_Ass_R1	GTTCTTGCTGCACCTGGTC	Internal fragment of
Mac_Ass_F1	CCTGTTTCACAGCTTCAAAGG	cargo DNA
Mac_Ass_F2	GCCTCACATTAATTGCGAATG	Internal fragment of
Mac_Ass_R2	AACACCTGCAGCCTAAATCG	cargo DNA
Pst DSM4166		
fasout_stz_1	GGTGAGAAATCAGATCAATGG	Round 1 *attI*
fasout_stz_2	GAATCACCGCTTCCTTGGTC	
fasout_stz_3	TGCTCAAGGTACTTCTTGGTC	Round 2 *attI*
fasout_stz_4	TGCTTGAAGTCACCGCGTAG	
fasbor_stz_1	CAGCAGCAGACGACACAGC	Round 1 *attB*
fasbor_stz_2	GCTGGACGTTGGCGTAGTC	
fasbor_stz_3	AGCCAGCGCATCGAAATC	Round 2 *attB*
fasbor_stz_4	GCGTACTTGTTGGTCAGCAC	
Ptz-RNA-F1	CCAGGGAAGAACGACAGGG	RT_PCR; amplicon 1
Ptz-RNA-R1	CCGAGTGCTCCATGGTGATG	Fig. 2
Ptz-RNA-FA	TCAACTAGCGGTGGTCAACC	RT_PCR; amplicon 2
FasInt1_R	TGCTTTCCAGCATCGTGTAG	Fig. 2
Ptz-RNA-F2	ACATGGATGTTCGGCTGGAG	RT_PCR; amplicon 3
Ptz-RNA-R2	GTTGACTGAATGACTGGCCG	Fig. 2
Ptz-RNA-FB	TCACGGGCACTTAGGATTCG	RT_PCR; amplicon 4
Ptz-RNA-Rb	GGCACATTCTCAATGGCTGC	Fig. 2
Ptz-RNA-F3	TGTACTCCAATCACCGAGCG	RT_PCR; amplicon 5
Ptz-RNA-R3	TGAAGCTACAGCCGTCGAAG	Fig. 2
Ptz-RNA_FC2	TGAGAGCCATGATCGCACTG	RT_PCR; amplicon 6
Ptz-RNA-RC2	AGAACTCTGATCGCGTCACG	Fig. 2
Ptz-RNA-F4	CGATAACGGATGGACGCTGG	RT_PCR; amplicon 7
FasInt4_R1	TCTGACCATTCCTCAATCAGC	Fig. 2
Ptz-RNA-FD	TCGCCAGTCCCTGATGAAAC	RT_PCR; amplicon 8
Ptz-RNA-RD	CATATCCGCATTGGCTTGCC	Fig. 2
Ptz-RNA-RD2	TTCATGGCTGGATCGGTCTG	RT_PCR; amplicon 9 Fig. 2 with Ptz-RNA-FD
RNA-stz-ABC-F	TCAAGATCGGCAACTACGAG	RT_PCR;
RNA-stz-ABC-R	GAGGAACCAGCGGTCGTG	ABC transporter
RNA-stz-rpoD-R	AGCACGGATCTGGTTCAGAG	Internal fragment of
RNA-stz-rpoD-F	GGACGACGACGAAGAAGAAG	*ropD*
RNA-stz-gyrb-R	TTGAAGCTGTCGTTCCACTG	Internal fragment of
RNA-stz-gyrb-F	ACGCAGATCCATTTCAAACC	*gyrB*
ginA-F	ACTAGTCAGTCGGTGCCCATGACTAT	Entire *ginA* gene.
ginA-R	ACTAGTGTTGACTGAATGACTGGCCG	Used for cloning

ginB-F	ACTAGTACATGGATGTTCGGCTGGAG	Entire *ginB* gene.
ginB-R	ACTAGTTGAAGCTACAGCCGTCGAAG	Used for cloning
ginC-F	ACTAGTTGTACTCCAATCACCGAGCG	Entire *ginC* gene.
ginC-R	ACTAGTTCTGACCATTCCTCAATCAGC	Used for cloning
ginD-F	ACTAGTCGATAACGGATGGACGCTGG	Entire *ginD* gene.
ginD-R	ACTAGTTTCATGGCTGGATCGGTCTG	Used for cloning
ginA_mutF	GCATCTGGCTGTGTTTGAGG	With Ptz-RNA-Rb for mutagenesis
ginB_mut_R	TTGGCAAACTCCGTGAAGGG	With Ptz-RNA-F2 for mutagenesis
ginD_mutR	GCCATTCGCACGATTACGAC	With Ptz-RNA-FC for mutagenesis

[a] *attI* and *attB* refer to the specific amplicons obtained from the circular intermediate and from the empty chromosomal site, respectively, resulting from excision of GInts. The *attI* of GInt7 in *P. cannabina* pv. alisalensis ES4326 was detected using primers fasout_pisi_2 and fasout_CYL_3 in the first round, and primers fasout_stz_3 and fasout_pisi_2 for the second round. Abbreviations are: Pfu, *P. fluorescens*; Ppu, *P. putida*; Pph, *P. syringae* pv. phaseolicola; Pca, *P. cannabina* pv. alisalensis; Pfr, *P. fragi*; Pto, *P. syringae* pv. tomato; Ppi, *P. syringae* pv. pisi; Pst, *P. stutzeri*.

[b] Unless specifically indicated, all primers were designed in this study.

[c] The SpeI adaptor of primers is underlined.

References

1. De Leij, F., Sutton, E. J., Whipps, J. M., Fenlon, J. S. & Lynch, J. M. Impact of Field Release of Genetically Modified Pseudomonas fluorescens on Indigenous Microbial Populations of Wheat. *Appl. Environ. Microbiol.* **61,** 3443-53 (1995).
2. Rico, A. *et al.* Nontoxigenic strains of *P. syringae* pv. *phaseolicola* are a main cause of halo blight of beans in Spain and escape current detection methods. *Phytopathology* **93,** 1553-1559 (2003).
3. Joardar, V. *et al.* Whole-genome sequence analysis of *Pseudomonas syringae* pv. phaseolicola 1448A reveals divergence among pathovars in genes involved in virulence and transposition. *J. Bacteriol.* **187,** 6488-6498 (2005).
4. Feil, H. *et al.* Comparison of the complete genome sequences of *Pseudomonas syringae* pv. syringae B728a and pv. tomato DC3000. *Proc. Natl. Acad. Sci.* **102,** 11064-9 (2005).
5. Martínez-García, P. M. *et al.* Bioinformatics analysis of the complete genome sequence of the mango tree pathogen *Pseudomonas syringae* pv. syringae UMAF0158 reveals traits relevant to virulence and epiphytic lifestyle. *PLoS ONE* **10,** e0136101, doi:10.1371/journal.pone.0136101 (2015).
6. Cuppels, D. A. & Ainsworth, T. Molecular and physiological characterization of *Pseudomonas syringae* pv. tomato and *Pseudomonas syringae* pv. maculicola strains that produce the phytotoxin coronatine. *Appl. Environ. Microbiol.* **61,** 3530-3536 (1995).
7. Buell, C. R. *et al.* The complete genome sequence of the *Arabidopsis* and tomato pathogen *Pseudomonas syringae* pv. tomato DC3000. *Proc. Natl. Acad. Sci.* **100,** 10181-10186, doi:10.1073/pnas.1731982100 (2003).
8. Prentki, P. & Krisch, H. M. In vitro insertional mutagenesis with a selectable DNA fragment. *Gene* **29,** 303-313 (1984).

9. Newman, J. R. & Fuqua, C. Broad-host-range expression vectors that carry the l-arabinose-inducible *Escherichia coli araBAD* promoter and the *araC* regulator. *Gene* **227,** 197-203, doi:10.1016/S0378-1119(98)00601-5 (1999).

10. Quandt, J. & Hynes, M. F. Versatile suicide vectors which allow direct selection for gene replacement in Gram-negative bacteria. *Gene* **127,** 15-21 (1993).

11. Schäfer, A. *et al.* Small mobilizable multi-purpose cloning vectors derived from the *Escherichia coli* plasmids pK18 and pK19: selection of defined deletions in the chromosome of *Corynebacterium glutamicum*. *Gene* **145,** 69-73, doi:http://dx.doi.org/10.1016/0378-1119(94)90324-7 (1994).

12. Giddens, S. R. *et al.* Mutational activation of niche-specific genes provides insight into regulatory networks and bacterial function in a complex environment. *Proc. Natl. Acad. Sci.* **104,** 18247-18252, doi:10.1073/pnas.0706739104 (2007).

13. Murillo, J. *et al.* Variation in conservation of the cluster for biosynthesis of the phytotoxin phaseolotoxin in *Pseudomonas syringae* suggests at least two events of horizontal acquisition. *Res. Microbiol.* **162,** 253-261, doi:10.1016/j.resmic.2010.10.011 (2011).
